# Association between 8q24 (rs13281615 and rs6983267) polymorphism and breast cancer susceptibility: a meta-analysis involving 117,355 subjects

**DOI:** 10.18632/oncotarget.12009

**Published:** 2016-09-13

**Authors:** Yafei Zhang, Xianling Zeng, Hongwei Lu, Hong Ji, Enfa Zhao, Yiming Li

**Affiliations:** ^1^ Department of General Surgery, The Second Affiliated Hospital of Xi'an Jiaotong University, Xi'an, Shaanxi, China; ^2^ Department of Obstetrics and Gynecology, The First Affiliated Hospital of Xi'an Jiaotong University, Xi'an, Shaanxi, China; ^3^ Department of Ultrasound, The Second Affiliated Hospital of Xi'an Jiaotong University, Xi'an, Shaanxi, China

**Keywords:** breast cancer, 8q24, rs13281615, rs6983267, meta-analysis

## Abstract

Published data on the association between 8q24 polymorphism and breast cancer (BC) risk are inconclusive. Thus, we conducted a meta-analysis to evaluate the relationship between 8q24 (rs13281615 and rs6983267) polymorphism and BC risk. We searched PubMed, EMBASE, Web of Science and the Cochrane Library up to August 13, 2015 for relevant studies. Odds ratios (ORs) and 95% confidence intervals (CIs) were used to estimate the strength of associations. Twenty-six studies published from 2008 to 2014, with a total of 52,683 cases and 64,672 controls, were included in this meta-analysis. The pooled results showed that there was significant association between 8q24 rs13281615 polymorphism and BC risk in any genetic model. In the subgroup analysis by ethnicity, the effects remained in Asians and Caucasians. However, no genetic models reached statistical association in Africans. There was no association in any genetic model in rs6983267. This meta-analysis suggests that 8q24 rs13281615 polymorphism is a risk factor for susceptibility to BC in Asians, Caucasians and in overall population, While, there was no association in Africans. The rs6983267 polymorphism has no association with BC risk in any genetic model. Further large scale multicenter epidemiological studies are warranted to confirm this finding.

## INTRODUCTION

Breast cancer (BC), one of the most frequently encountered malignant tumors in women, has become the main reason of tumor-associated death in our word [1], whose incidence rate is increasing year by year and accompanied by younger trend in the world [2, 3]. The etiology of BC is very complex, many factors cause it's occurrence and development, the specific mechanism has not been clarified. Epidemiology and basic etiology studies have shown that BC's occurrence and progression is closely related to multiple factors interaction between genetic, endocrine and external environment [4-6].

The genetic markers of BC are mainly expressed in two forms, that is, the higher external rate mutation gene and the lower external rate polymorphism gene. Compared with familial BC, most sporadic BC is associated with lower external rate polymorphism genes [7, 8]. The distribution of these genes in the population is more than 1%, and the genetic variation of these genes (Single nucleotide polymorphism, SNP) is mainly by changing the expression level and function of the protein and the modified factor inside and outside the cell, thus affecting the genetic susceptibility of BC [8]. In recent years, many BC-associated gene loci have been demonstrated by GWAS, 8q24 (rs13281615 and rs6983267) is the gene mutation site selected by this technique [7].

Previous functional studies have reported the association between 8q24 (rs13281615 and rs6983267) polymorphism and BC risk [9-29]. Because of differences in race and region, studies of this site are not entirely consistent with the conclusion that whether the site is associated with the risk of BC. To clarify the role of 8q24 (rs13281615 and rs6983267) polymorphism in BC risk, five meta-analysis [30-34] on the associations between 8q24 (rs13281615 and rs6983267) polymorphism and BC had been carried out. However, the results remain inconclusive and number of their studies included in their meta-analysis about BC is small. In the subgroup of their analyses the sample size is extremely small, and some just no subgroup. Therefore, we decided to carry out this meta-analysis on all the included case-control researches to make a more accurate assessment of the relationship. Moreover, we performed a subgroup analysis stratified by ethnicity.

## RESULTS

### Characteristics of included papers

**Figure 1:**
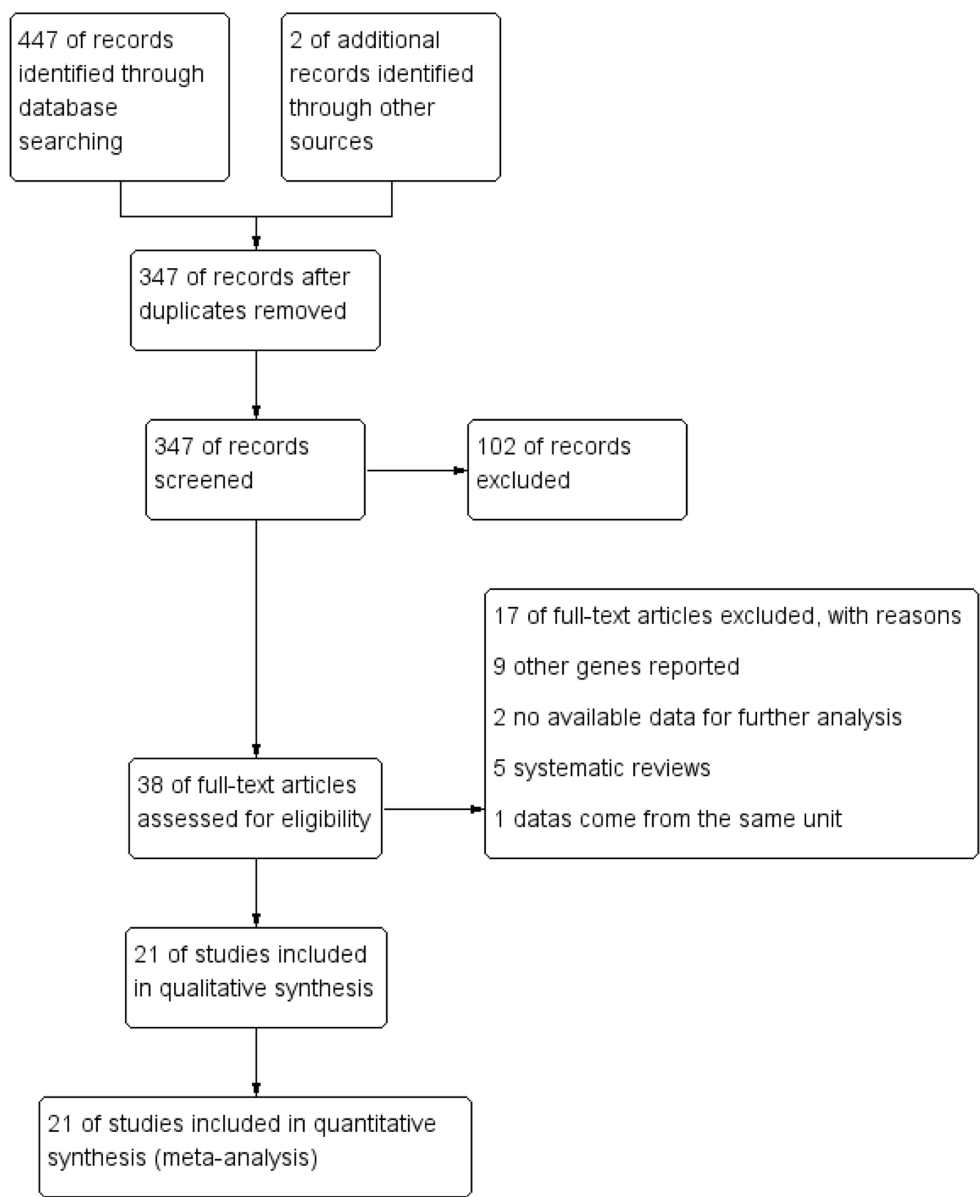
Flow chart of studies selection in this meta-analysis

The specific search process is shown in Figure 1. A total of 447 references were preliminarily identified at first based on our selection strategy. We also identified 2 papers [11, 29] through other source. 347 records left after removing repeated studies. We refer to titles or abstracts of all the included literatures, and then removed obviously irrelevant papers. In the end, the whole of the rest of the papers were checked based on the inclusion and exclusion criteria. Finally, 21 studies [9-29] on 8q24 (rs13281615 and rs6983267) polymorphism and the occurrence of BC were eventually included in our study, including 52,683 cases and 64,672 controls. Characteristics of eligible analysis are shown in Table 1. The 21 case-control papers were published between 2008 and 2014, among them, 1 study was performed in African, 7 in Asian, 12 in Caucasians and 1 in both African and Caucasians. All studies were case-controlled.

**Table 1:** Characteristics of the studies included in the meta-analysis

First author	Year	Country	Ethnicity	Source of controls	Number(case/control)	HWE
rs13281615						
Antoniou[9]	2009	Mixed	Caucasians	Nested	7787/6662	0.100
Bai[10]	2014	China	Asian	PB	280/287	0.086
Barnholtz-Sloan[11]	2010	United States	Caucasians	PB	1223/1117	0.220
Barnholtz-Sloan[11]	2010	United States	African	PB	736/658	0.580
Campa[12]	2011	USA and Europe	Caucasians	PB	8302/11615	0.106
Chan[13]	2012	Singapore	Asian	PB	1174/1463	0.047
Elematore[14]	2014	Chile	Caucasians	PB	347/801	0.993
Fletcher[15]	2008	United Kingdom	Caucasians	PB	1470/1341	0.366
Garcia-Closas[16]	2008	Mixed	Caucasians	Nested	15084 /22105	0.672
Gorodnova[17]	2010	Russian	Caucasians	PB	140/174	0.710
Harlid[18]	2012	European	Caucasians	PB	3545/5007	0.045
Jiang[19]	2011	China	Asian	PB	493/510	1.000
Latif[20]	2010	British	Caucasians	HB	919/343	0.639
Li[29]	2011	China	Asian	HB	558/635	0.748
Long[21]	2010	China	Asian	PB	2945/2981	0.985
McInerney[22]	2009	Ireland	Caucasians	PB	917/993	0.096
Mizoo[23]	2012	Japan	Asian	PB	466/458	0.252
Shan[24]	2012	Tunisia	African	PB	639/365	0.497
Tamimi[25]	2010	Sweden	Caucasians	PB	661/711	<0.001
Teraoka[26]	2011	Denmark and USA	Caucasians	PB	606/1194	0.041
Zhang[28]	2014	China	Asian	HB	482/527	0.089
rs6983267						
Fletcher[15]	2008	United Kingdom	Caucasians	PB	1480/1336	0.453
McInerney[22]	2009	Ireland	Caucasians	PB	945/957	0.349
Wokolorczyk[27]	2008	Canada	Caucasians	PB	1006/1910	0.266
Zhang[28]	2014	China	Asian	HB	478/522	0.046

HWE: Hardy-Weinberg equilibrium; PB: population based; HB: hospital-based; SNP: single nucleotide polymorphism.

### Meta-analysis results

Table 2 and Table 3 shows the 8q24 (rs13281615 and rs6983267) polymorphisms genotype distribution and allele frequencies in in case group and control group. Main results of our study were shown in Table 4. A total of 21 studies with 52,683 cases and 64,672 controls were included. As shown in Table 4, The pooled results indicated that the correlation between 8q24 rs13281615 polymorphism and the occurrence of BC was significant in any genetic model: Allele model (OR = 1.10, 95% CI = 1.07-1.13, *P* < 0.00001), dominant model (OR = 1.13, 95% CI = 1.08-1.18, *P* < 0.00001) recessive model (OR = 1.13, 95% CI = 1.08-1.18, *P* < 0.00001) homozygous genetic model (OR = 1.20, 95% CI = 1.13-1.27, *P* < 0.00001) heterozygote comparison (OR = 1.09, 95% CI = 1.06-1.12, *P* < 0.00001).

**Table 2:** rs13281615 polymorphisms genotype distribution and allele frequency in cases and controls

First author	Genotype (N)								Allele frequency (N)			
	Case				Control				Case		Control	
	Total	GG	AG	AA	Total	GG	AG	AA	G	A	G	A
Antoniou	7787	1396	3872	2519	6662	1158	3317	2187	6664	8910	5633	7691
Bai	280	63	152	65	287	62	158	67	278	282	282	292
Barnholtz-Sloan	1223	230	604	389	1117	194	519	404	1064	1382	907	1327
Barnholtz-Sloan	736	130	387	219	658	123	331	204	647	825	577	739
Campa	8302	1764	4044	2494	11615	2193	5609	3813	7572	9032	9995	13235
Chan	1174	317	554	303	1463	364	693	406	1188	1160	1421	1505
Elematore	347	131	148	68	801	255	394	152	410	284	904	698
Fletcher	1470	305	730	435	1341	225	629	487	1340	1600	1079	1603
Garcia-Closas	15084	2921	7284	4879	22105	3773	10682	7650	13126	17042	18228	25982
Gorodnova	140	42	63	35	174	38	84	52	147	133	160	188
Harlid	3545	719	1723	1103	5007	884	2357	1766	3161	3929	4125	5889
Jiang	493	125	247	121	510	127	255	128	497	489	509	511
Latif	919	185	464	270	343	56	160	127	834	1004	272	414
Li	558	162	285	111	635	173	313	149	609	507	659	611
Long	2945	796	1470	679	2981	745	1491	745	3062	2828	2981	2981
McInerney	917	178	467	272	993	182	456	355	823	1011	820	1166
Mizoo	466	180	211	75	458	177	206	75	571	361	560	356
Shan	639	201	303	135	365	96	176	93	705	573	368	362
Tamimi	661	223	263	175	711	273	277	161	709	613	823	599
Teraoka	606	140	292	174	1194	213	623	358	572	640	1049	1339
Zhang	482	143	248	91	527	124	283	120	534	430	531	523

**Table 3:** rs6983267 polymorphisms genotype distribution and allele frequency in cases and controls

First author	Genotype (N)								Allele frequency (N)			
	Case				Control				Case		Control	
	Total	GG	AG	AA	Total	GG	AG	AA	G	A	G	A
Fletcher	1480	338	734	408	1336	312	653	371	1410	1550	1277	1395
McInerney	945	230	464	251	957	245	464	248	924	966	954	960
Wokolorczyk	1006	254	507	245	1910	513	977	420	1015	997	2003	1817
Zhang	478	151	218	109	522	183	233	106	520	436	599	445

**Table 4:** Meta-analysis results

Comparisons	OR	95%CI	*P* value	Heterogeneity		Effects model
				I^2^	*P* value	
**Allele model**						
**rs13281615**	**1.10**	**1.07-1.13**	***P* < 0.00001**	**49%**	**0.006**	**R**
African	1.08	0.96-1.22	0.18	58%	0.12	F
**Asian**	**1.08**	**1.03-1.14**	**0.001**	**0%**	**0.77**	**F**
**Caucasians**	**1.11**	**1.06-1.16**	***P* < 0.00001**	**67%**	**0.0004**	**R**
rs6983267	0.95	0.89-1.01	0.10	0%	0.66	F
Dominant model						
**rs13281615**	**1.13**	**1.08-1.18**	***P* < 0.00001**	**39%**	**0.03**	**R**
African	1.13	0.94-1.36	0.18	0%	0.34	F
**Asian**	**1.11**	**1.03-1.21**	**0.009**	**0%**	**0.92**	**F**
**Caucasians**	**1.14**	**1.07-1.21**	***P* < 0.0001**	**63%**	**0.002**	**R**
rs6983267	0.94	0.85-1.04	0.24	0%	0.63	F
Recessive model						
**rs13281615**	**1.13**	**1.08-1.18**	***P* < 0.00001**	**30%**	**0.10**	**R**
African	1.09	0.89-1.32	0.40	60%	0.11	F
**Asian**	**1.11**	**1.03-1.20**	**0.008**	**0%**	**0.79**	**F**
**Caucasians**	**1.15**	**1.08-1.22**	***P* < 0.00001**	**50%**	**0.02**	**R**
rs6983267	0.93	0.84-1.03	0.15	0%	0.89	F
Homozygous genetic model						
**rs13281615**	**1.20**	**1.13-1.27**	***P* < 0.00001**	**43%**	**0.02**	**R**
African	1.16	0.92-1.47	0.22	60%	0.12	F
**Asian**	**1.17**	**1.07-1.29**	**0.001**	**0%**	**0.77**	**F**
**Caucasians**	**1.22**	**1.12-1.32**	***P* < 0.00001**	**62%**	**0.002**	**R**
rs6983267	0.90	0.80-1.02	0.10	0%	0.69	F
Heterozygote comparison						
**rs13281615**	**1.09**	**1.06-1.12**	***P* < 0.00001**	**22%**	**0.17**	**F**
African	1.12	0.93-1.36	0.24	0%	0.68	F
**Asian**	**1.08**	**1.00-1.18**	**0.06**	**0%**	**0.98**	**F**
**Caucasians**	**1.11**	**1.05-1.17**	**0.0005**	**55%**	**0.01**	**R**
rs6983267	0.96	0.86-1.07	0.45	0%	0.74	F

F-fixed effects model; R-random effects model.

The subgroup study stratified by ethnicity showed an increased BC risk in Asians (Allele model: OR = 1.08, 95% CI = 1.03-1.14, *P* = 0.001; dominant model: OR = 1.11, 95% CI = 1.03-1.21, *P* = 0.009; recessive model: OR = 1.11, 95% CI = 1.03-1.20, *P* = 0.008; homozygous genetic model: OR = 1.17, 95% CI = 1.07-1.29, *P* = 0.001; heterozygote comparison: OR = 1.08, 95% CI = 1.00-1.18, *P* = 0.06) and Caucasians (Allele model: OR = 1.11, 95% CI = 1.06-1.16, *P* < 0.00001; dominant model: OR = 1.14, 95% CI = 1.07-1.21, *P* < 0.0001; recessive model: OR = 1.15, 95% CI = 1.08-1.22, *P* < 0.00001; homozygous genetic model: OR = 1.22, 95% CI = 1.12-1.32, *P* < 0.00001; heterozygote comparison: OR = 1.11, 95% CI = 1.05-1.17, *P* = 0.0005). While, there was not any genetic model attained statistical correlation in Africans. There was no association in any genetic model in rs6983267 (Table 4).

### Sensitivity analyses

As shown in Table 1, all the studies conformed to he balance of Hardy-Weinberg equilibrium (HWE) in controls except 5 studies (*P* < 0.05), however, after performing the sensitivity analyses, the overall outcomes were no statistically significant change when removing any of the articles, indicating that our study has good stability and reliability.

### Detection for heterogeneity

Heterogeneity among studies was obtained by *Q* statistic. Random-effect models were applied if *p*-value of heterogeneity tests were less than 0.1 (*p* ≤ 0.1), otherwise, fixed-effect models were selected (Table 4).

### Publication bias

**Figure 2:**
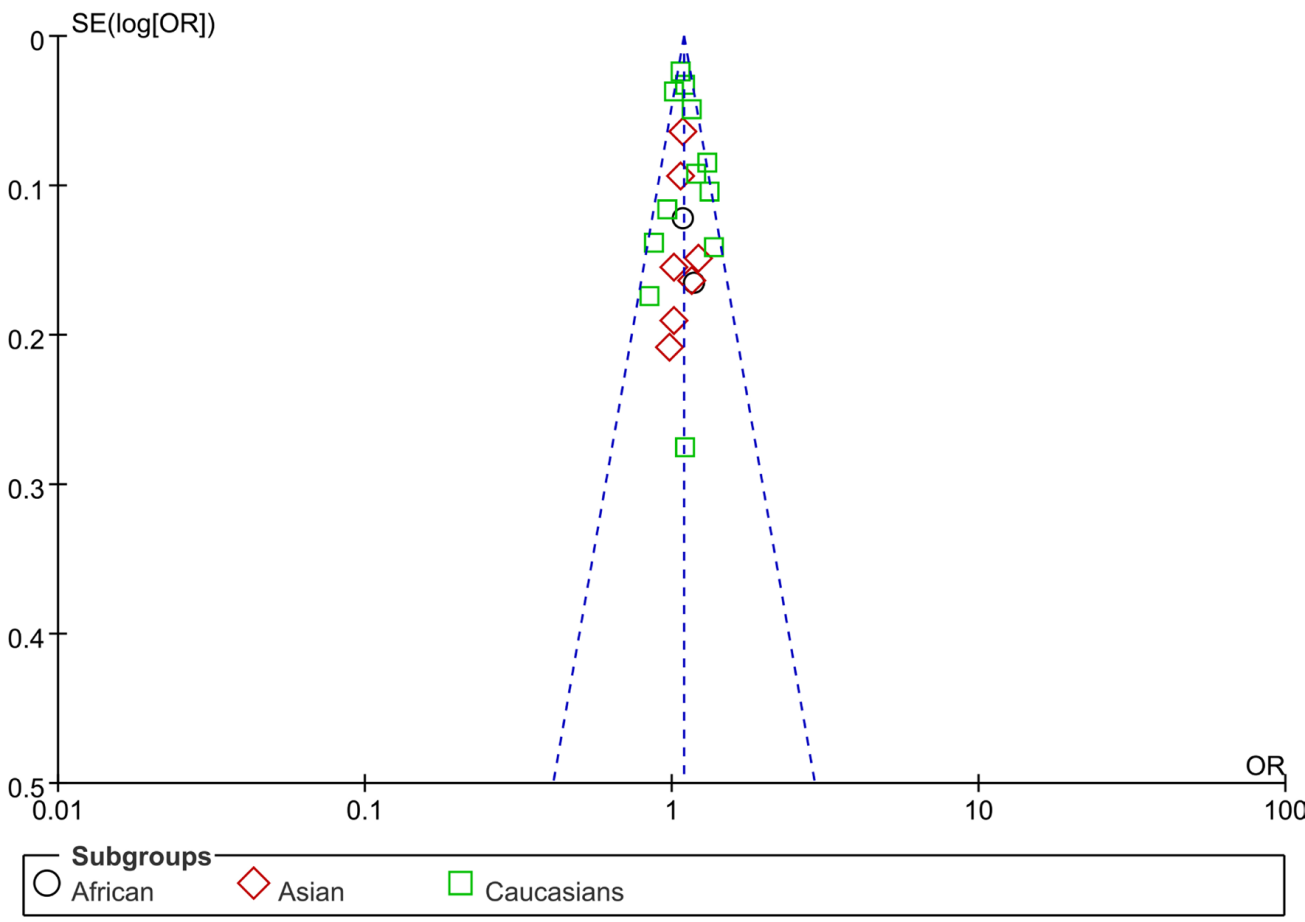
Funnel plot on the association of rs13281615 variant and breast cancer in a fixed-effect model (heterozygote comparison). Abbreviations: SE, standard error; OR, odds ratio; A *vs*. G, Allele model.

**Figure 3:**
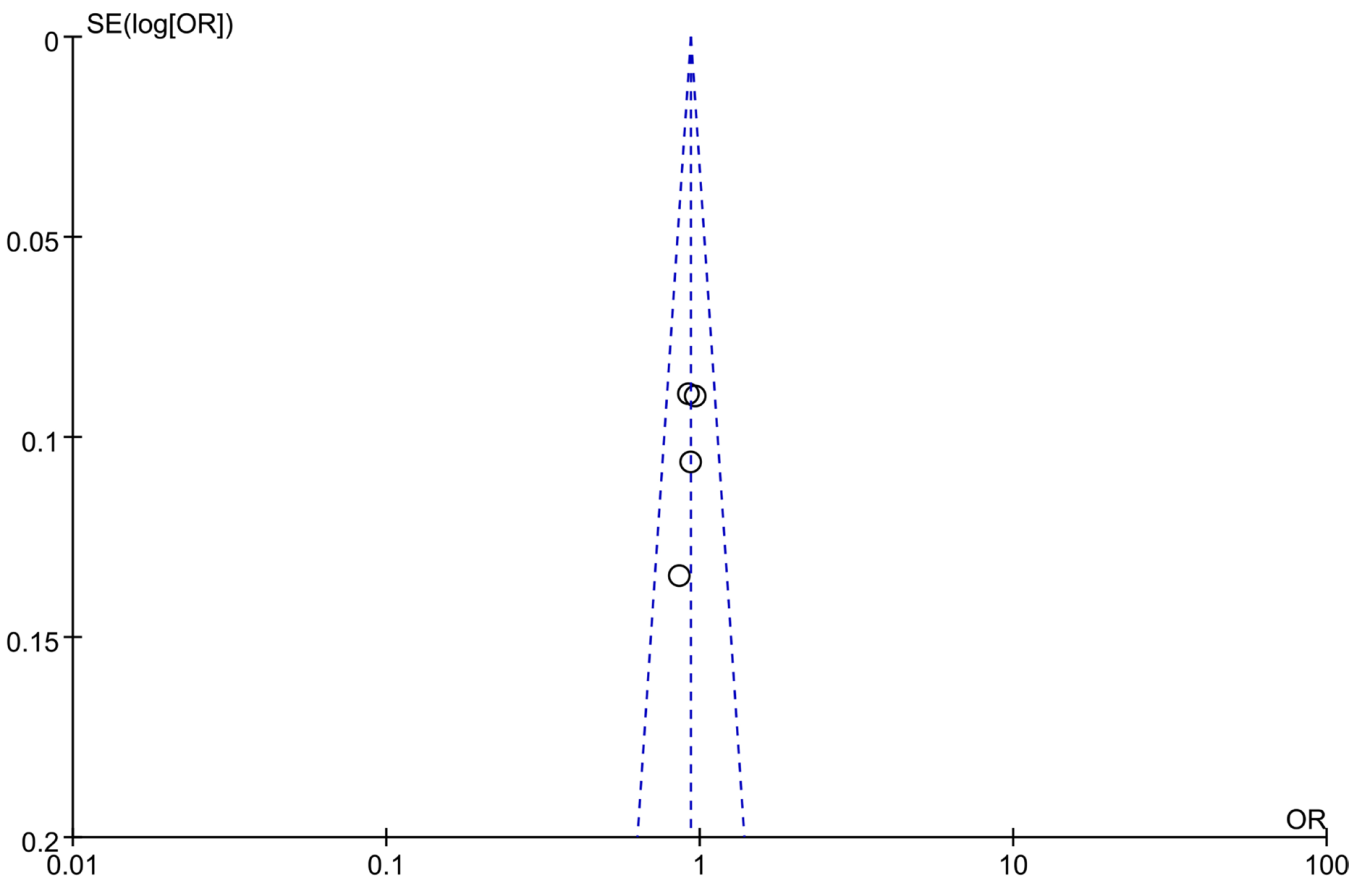
Funnel plot on the association of rs6983267 variant and breast cancer in a fixed-effect model (recessive model). Abbreviations: SE, standard error; OR, odds ratio; A vs. G, Allele model.

We use Begg's funnel plot and Egger test to evaluate the published bias. As Figure 2 (rs13281615) and Figure 3 (rs6983267) indicated, the funnel plot is symmetrical, indicating that there is no significant publication bias in the total population. In our study, no significant publication bias was found in the Begg's test and Egger's test (*P* > 0.05).

## DISCUSSION

A large amount of evidence suggests that genetics is important in determining the risk of cancer [35]. Related research is to search for the susceptibility genes associated with cancer. It is believed that SNP is the main cause of human genetic variation, which may increase individual risk to suffer cancer [14, 18, 22, 23]. With the development of medical science, hereditary susceptibility to cancer has caused people's great interest, and the study on the genetic polymorphism of the tumor is increasing.

At present, BRCA1 and BRCA2 gene polymorphisms are generally considered to be associated with BC, but because of the low mutation rate, it can only be detected in the small part of BC patients [36]. In 2007, the whole genome association analysis identified many genetic polymorphisms that may be associated with the development of BC, which included rs6983267 and rs13281615 [37].

The 8q24 rs6983267 and rs13281615 bit is located in the non - base region of the dye - color 8q24, and Its function is not very clear. 8q24.12-24.13 location myelocytomatosis oncogene (MYC), MYC is a group of cancer genes, which are not expressed in normal cells, and expressed in some cancer cells. MYC can promote cell proliferation, immortalized, differentiation and transformation. It is an significant factor in the formation of BC, colon cancer and prostate cancer [38, 39]. Studies shown that the chromosome 8q24 region is related to the occurrence of prostate cancer and colon cancer, which can play a role in carcinogenesis through interacting with MYC gene amplification and over expression [40]. The change of some loci of a single gene may have limited impact on the ultimate effect of cancer, and it is needed to carry out large-scale studies in populations.

Recently, a growing number of epidemiological studies have been carried out to explore the relationship between 8q24 (rs13281615 and rs6983267) polymorphisms and the occurrence of BC. Nevertheless, the conclusions are still inconclusive. Therefore, we carried out the meta-analysis on the whole included case-control researches to make a more accurate assessment of the relationship.

In our study, 21 studies were eventually included, including 52,683 cases and 64,672 controls. And we assessed the relationship between 8q24 (rs13281615 and rs6983267) polymorphisms and the occurrence of BC. In the total population, the pooled results indicated that the correlation between 8q24 rs13281615 polymorphism and the occurrence of BC was significant in any genetic model (Table 4). It was partially consistent with the consequences of previous five meta-analysis [30-34], while the sample size was several times than theirs, make the results more convincing.

BC is a disease with a difference in the incidence and mortality among different ethnic groups. Each increase of 25% European descent, the risk of developing BC will increase by 1.79 times [41]. Easton [42] et al believes that the incidence of the European population is higher than that of other ethnic groups, which are consistent with the results of this study. The subgroup study stratified by ethnicity showed an increased BC risk in Asians and Caucasians (Table 4). While, there was not any genetic model attained statistical correlation in Africans. There was no association in any genetic model in rs6983267 (Table 4). It was partially consistent with the Dai(2013)'s study [30] (with 11 papers containing 40,762 cases and 50,380 controls) and Pei(2013)'s [32] (with 12 eligible studies including 42,508 cases and 53,928 controls) findings. Another meta-analysis by Song(2012) [33] et al, including seven papers with 22,128 cases and 29,276 controls, they found no relationship between rs13281615 polymorphism and BC susceptibility in Asians. This contradiction may be caused by sample sizes and racial differences.

Our meta-analysis has some limitations in the following aspects. First, our study is a summary of the data. Due to the lack of the original data needed, we could not evaluate the cancer susceptibility stratified by age, Sex, environment, hormone level, menopause age and other risk factors. We also cannot analyze these studies to analyze the potential interaction of gene - environment and gene - gene. Secondly, we just included the published papers in our study, there may still be some published studies in line with the conditions. Moreover, our study is a summary of the data. We did not verify it from the level of basic experiments. Data from a large sample of multiple centers is still needed to confirm the relationship between 8q24 (rs13281615 and rs6983267) polymorphisms and BC risk.

In summary, our study suggests that 8q24 rs13281615 polymorphism could increase the risk of BC in Asians, Caucasians and in overall population, While, there was no association in Africans. The rs6983267 polymorphism has no relationship with the occurrence of BC in any genetic model. Data from a large sample of multiple centers is still needed to confirm our findings.

## MATERIALS AND METHODS

### Literature searching strategy

We searched PubMed, EMBASE, Web of science, the Cochrane Library for relevant studies published before August 13, 2015. The following keywords were used: rs6983267/rs13281615/8q24, variant*/genotype/polymorphism/SNP, breast AND (cancer or carcinom* or neoplasm* or tumor) and the combined phrases for all genetic studies on the association between the 8q24 (rs13281615 and rs6983267) polymorphism and BC risk. The reference lists of all articles were also manually screened for potential studies. Abstracts and citations were screened independently by two authors, all the agreed articles need a second screen for full-text reports. The searching was done without restriction on language.

### Selection and exclusion criteria

Inclusion criteria: A study was included in this meta-analysis if it met the following criteria: i) independent case-control studies for humans; ii) the study evaluated the association between 8q24 (rs13281615 and rs6983267) polymorphism and BC risk; iii) has available genotype frequencies in cancer cases and control subjects for risk estimate. We excluded comments, editorials, systematic reviews or studies lacking sufficient data. If the publications were duplicated or shared in more than one study, the most recent publications were included. All identified studies were screened by two investigators independently. What's more, there was no limitation for publication language.

### Data extraction and synthesis

We used endnote bibliographic software to construct an electronic library of citations identified in the literature search. All the PubMed, EMBASE, Web of science and the Cochrane Library searches were performed using Endnote; duplicates were found automatically by endnote and deleted manually. All data extraction were checked and calculated twice according to the inclusion criteria listed above by two independent investigators. Data extracted from the included studies were as follows: First author, year of publication, country, ethnicity, Source of controls, Genotyping method, number of cases and controls and evidence of HWE in controls. A third reviewer would participate if some disagreements were emerged, and a final decision was made by the majority of the votes.

### Statistical analysis

All statistical analyses were performed using STATA version 11.0 software (StataCorp LP, College Station, TX) and Review Manage version 5.2.0 (The Cochrane Collaboration, 2012). Hardy-Weinberg equilibrium (HWE) was assessed by χ2 test in the control group of each study [43]. The strength of associations between the 8q24 (rs13281615 and rs6983267) polymorphism and BC risk were measured by odds ratio (ORs) with 95% confidence interval (CIs). *Z* test was used to assess the significance of the ORs, *I*^2^ and *Q* statistics was used to determine the statistical heterogeneity among studies. A random-effect model was used if p value of heterogeneity tests was no more than 0.1 (*p* ≤ 0.1), otherwise, a fixed-effect model was selected [43, 44]. Sensitivity analyses were performed to assess the stability of the results. We used Begg's funnel plot and Egger's test to evaluate the publication bias [45, 46]. The strength of the association was estimated in the allele model, the dominant model, the recessive model, the homozygous genetic model, and the heterozygous genetic model, respectively. *p* < 0.05 was considered statistically significant. We performed subgroup according to Ethnicity.
